# Sampling rare conformational transitions with a quantum computer

**DOI:** 10.1038/s41598-022-20032-x

**Published:** 2022-09-29

**Authors:** Danial Ghamari, Philipp Hauke, Roberto Covino, Pietro Faccioli

**Affiliations:** 1 Department of Physics, University of Trento, Via Sommarive 14, Trento, 38123 Italy; 2 INFN-TIFPA, Via Sommarive 14, Trento, 38123 Italy; 3 INO-CNR BEC Center & Department of Physics, University of Trento, Via Sommarive 14, Trento, 38123 Italy; 4 Frankfurt Institute for Advanced Studies, Ruth-Moufang-Straße 1, Frankfurt am Main, 60438 Germany

**Keywords:** Physics, Biological physics, Information theory and computation, Statistical physics, thermodynamics and nonlinear dynamics, Physical chemistry

## Abstract

Structural rearrangements play a central role in the organization and function of complex biomolecular systems. In principle, Molecular Dynamics (MD) simulations enable us to investigate these thermally activated processes with an atomic level of resolution. In practice, an exponentially large fraction of computational resources must be invested to simulate thermal fluctuations in metastable states. Path sampling methods focus the computational power on sampling the rare transitions between states. One of their outstanding limitations is to efficiently generate paths that visit significantly different regions of the conformational space. To overcome this issue, we introduce a new algorithm for MD simulations that integrates machine learning and quantum computing. First, using functional integral methods, we derive a rigorous low-resolution spatially coarse-grained representation of the system’s dynamics, based on a small set of molecular configurations explored with machine learning. Then, we use a quantum annealer to sample the transition paths of this low-resolution theory. We provide a proof-of-concept application by simulating a benchmark conformational transition with all-atom resolution on the D-Wave quantum computer. By exploiting the unique features of quantum annealing, we generate uncorrelated trajectories at every iteration, thus addressing one of the challenges of path sampling. Once larger quantum machines will be available, the interplay between quantum and classical resources may emerge as a new paradigm of high-performance scientific computing. In this work, we provide a platform to implement this integrated scheme in the field of molecular simulations.

## Introduction

Molecular dynamics (MD) simulations enable us to investigate the structure and dynamics of molecular systems at high spatial and temporal resolution^1^. Despite their large success, MD simulations face the challenge of sampling rare thermally activated re-organizations of complex systems, e.g., conformational changes, folding, and phase transitions^2^. Indeed, in a typical simulation, an exponentially large fraction of the computational time is employed to simulate thermal fluctuations in meta-stable states, rather than sampling the fast transition paths—the stochastic jumps between states—which are rare events^2^.

A wide spectrum of clever enhanced sampling methods have been developed over the last two decades to overcome the rare events sampling problem^3^. Some of these techniques reach a high computational efficiency by introducing history-dependent biasing forces that drive the system out of its thermal equilibrium, thus promoting the escape from meta-stable states (see, e.g.,^4–7^). The biasing forces depend on collective variables (CVs), which should encode the essential low-dimensional features of a molecular rare event^8^. In practice, identifying optimal CVs is a very hard problem, and in realistic conditions sub-optimal CVs will affect the quality of the sampling and the accuracy of the mechanistic understanding emerging from the simulations^8^.

**Figure 1:**
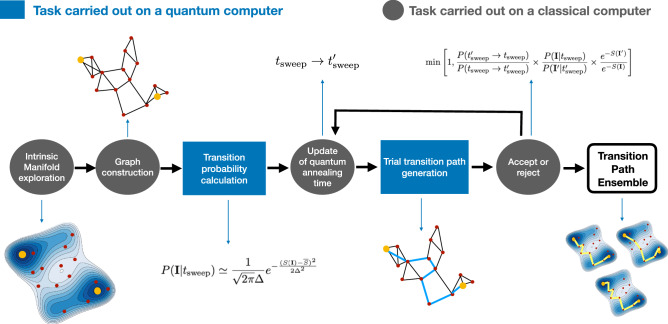
Schematic representation of the path sampling scheme introduced in this work, which combines ML and MD performed on a classical computer with QC performed on a quantum annealing machine. Our scheme samples the full transition path ensemble without any use of CVs or unphysical biases.

As an alternative approach, Transition Path Sampling (TPS)^9^ is a Markov Chain Monte Carlo scheme that in principle samples the transition path ensemble without involving any biasing force, nor a choice of CVs. In TPS, plain MD simulations generate a trial move, i.e., the attempt to generate a new transition path. For instance, in the so-called shooting move, a new trajectory is initialized from a configuration randomly selected from the last stored transition path^10^. Yet, when applied to complex transitions occurring in large configuration spaces with rugged energy landscapes, TPS faces two challenges: efficiently generating viable trial trajectories at an acceptable computational cost and reducing the correlation of generated paths^11^.

Even though promising advancements have recently been made by integrating molecular simulations with machine learning (ML) (see, e.g.,^12–18^), the quest for computationally affordable and accurate enhanced sampling of complex molecular systems remains open. In this endeavor, rapid advances in quantum computing provide new opportunities, as is illustrated in the context of quantum chemistry and biology by pioneering applications^19–26^. Over the last few years, quantum hardware has grown exponentially both in size and performance^27–29^, to a point that it is now realistic to foresee the onset of a tangible quantum advantage in computational problems^30,31^. It is therefore both important and timely to address the question whether MD, ML, and Quantum Computing (QC) can join forces to tackle outstanding challenges of molecular simulations.

In this work, we integrate MD, ML, and QC to sample the transition path ensemble of thermally activated rare events without involving any unphysical bias or choice of CVs. The salient features of this scheme are illustrated in Fig. 1: First, ML and MD perform a preliminary uncharted exploration of the most visited regions of the configuration space^32^. These data are used to derive a general coarse-grained description of rare events based on Langevin dynamics. Then, QC on a quantum annealing machine^33–37^ generates transition paths connecting the previously generated configurations. These paths are then accepted or rejected according to a Metropolis criterion implemented on a classical computer, which combines the statistical mechanics of the transition path ensemble with the internal physics of the quantum annealing machine, for which we used the D-Wave machine^38^.

Importantly, at each iteration the quantum computer generates a new viable and uncorrelated transition path. In fact, at every step the quantum computer is re-initialized in an equal superposition of all computational basis states, which erases all memory of the previously sampled transition paths. In this way, we harness one of the defining features of quantum annealing to overcome a key limitation of path sampling algorithms. In addition, we exploit non-adiabatic effects in the annealing procedure (controlled by the sweep time) and the presence of classical fluctuations (due to intrinsic noise of the physical machine) to explore the transition path ensemble.

As a first illustrative application, we sample the transition path ensemble of a conformational transition in alanine dipeptide. Even though our approach is fully general and scalable, we have chosen this benchmark system as it is sufficiently small to enable us to encode and run our algorithm on presently existing D-Wave machines, yet it recapitulates the features of rare events in molecular systems. Our benchnmark results agree well with those obtained by plain MD. We use an auto-correlation analysis to demonstrate that the quantum computer generates uncorrelated trial transition paths at every Monte Carlo step. Though existing quantum machines permit us to perform benchmarks only on proof-of-concept systems of limited complexity, the ongoing exponential growth in size and efficiency of quantum computing hardware suggests that, in the future, our approach might help us to investigate transitions that are currently challenging for state-of-the-art classical sampling methods.

The manuscript is organized as follows. In "Uncharted exploration of the intrinsic manifold" and "Coarse-grained representation of the dynamics on the intrinsic manifold" Section, we introduce the general theoretical framework, describe the algorithm used to perform the uncharted exploration of the intrinsic manifold, and our coarse-grained description of reactive processes. In 'Transition Path Sampling with a quantum annealer' Section, we discuss the encoding of the path sampling problem on a quantum annealing machine and derive our hybrid Monte Carlo scheme that combines classical and quantum computing. In 'Application to a molecular benchmark system' Section, we report on our illustrative application to alanine dipeptide. The main results are summarized and discussed in "Conclusions" Section.

## Theoretical setup

In a molecular system at thermal equilibrium, the statistically relevant configurations accumulate in low-dimensional regions that define the so-called intrinsic manifold. Our path sampling algorithm exploits a recently developed scheme to efficiently explore this intrinsic manifold^32^ (step 1 in Fig. 1). Then, it relies on a coarse-grained representation of the dynamics that is defined directly on this manifold, based on the configurations generated during the exploration, to define the input parameters for the quantum annealing part of our algorithm, discussed in "Transition Path Sampling with a quantum annealer" Section (step 2 in Fig. 1).

### Uncharted exploration of the intrinsic manifold.

To efficiently explore the intrinsic manifold and sample relevant molecular configurations without any use of CVs or nonphysical bias, we rely on Intrinsic Map Dynamics (iMapD)^32^. In iMapD, a data-driven manifold learning technique—diffusion maps^39^—empowers the unbiased MD sampling. Given some local sampling, diffusion maps identify the boundary of the explored configuration space in a low-dimensional representation. New unexplored configurations in the vicinity beyond this boundary are identified, from which we start a further round of local unbiased sampling. By iterating between these two steps, iMapD rapidly explores the relevant parts of the configuration space. In the sections S1 and S3 of the Supplementary Information (SI), we summarize the key aspects of the theory and implementation of iMapD.

Using iMapD we obtain a sparse data set of configurations ${\mathscr {C}}=\{Q_k\}_{k=1,\ldots ,\nu }$, that by construction lie on the intrinsic manifold, and that were obtained at a much lower computational cost than by running equilibrium MD^32^.

**Figure 2:**
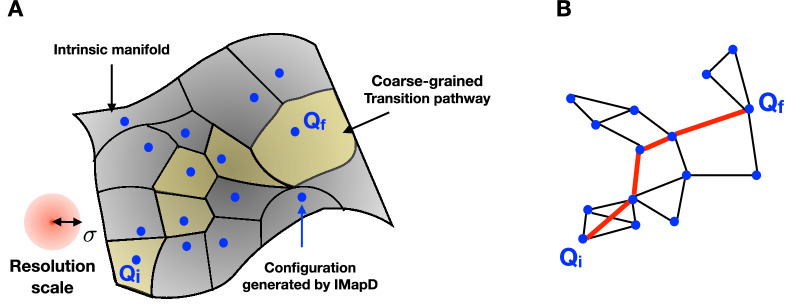
(**a**) Illustration of the coarse-grained representation of the Langevin dynamics. Dots represents configurations generated using iMapD, which lie on the intrinsic manifold. Each such configuration is regarded as a representative element of its Voronoi cell. Coarse-grained trajectories are identified by sequences of Voronoi cells, *I*. A typical transition path is highlighted in yellow. The intrinsic resolution scale $\sigma$ of the effective theory is set by the average distance between the configurations generated by iMapD. (**b**) Graph representation of the coarse-grained Langevin dynamics. The red line denotes the transition path highlighted in yellow in panel (**a**).

### Coarse-grained representation of the dynamics on the intrinsic manifold

Once the data set ${\mathscr {C}}$ is established, we can use it to build a coarse-grained representation of the dynamics, defined directly on the intrinsic manifold explored with iMapD.

The sparse configuration data set obtained after the uncharted exploration defines a partition of the intrinsic manifold in finite sub-regions. The *i*-th region is identified with the neighborhood of configuration $Q_i\in {\mathscr {C}}$. For example, in a Voronoi tassellation, $Q_i$ would represent the center of a cell whose boundaries lie midway to the neighboring sampled configurations (see Fig. 2).

The spatial resolution scale $\sigma$ of this coarse-grained representation of the molecular dynamics is set by the average distance between neighboring configurations in the data set ${\mathscr {C}}$. The temporal resolution $\Delta t$ is estimated by the average time the system takes to diffuse across neighboring regions. At this level of coarse-graining, transition pathways correspond to ordered sequences of visited sub-regions (see, e.g., the yellow regions in Fig. 2). Therefore, a transition path can be specified by an integer vector $\mathbf{I}= (i_1, \ldots , i_{N_I})$, where $i_k$ is the label pointing at the neighborhood of the configuration $Q_{i_k}$, which is visited at the *k*-th time step.

We develop a statistical mechanical formalism that enables us to describe the coarse-grained dynamics on the intrinsic manifold. This is necessary to correctly evaluate the probability of each coarse-grained transition path. We employ a powerful path-integral formalism combined with regularization and renormalization procedures that were originally developed in the framework of nuclear and subnuclear physics (see^40^ for an enlightening pedagogical introduction).

*Path integral formulation of stochastic dynamics* Assuming a diffusive dynamics, we derived an expression for the probability of arbitrary coarse-grained paths $\mathbf{I}$ on the intrinsic manifold, in the form

1 $$\begin{aligned} P(\mathbf{I})\propto & {}\, e^{-S(\mathbf{I})}, \end{aligned}$$

where the functional $S(\mathbf{I})$ is called the effective action of the coarse-grained path $\mathbf{I}$. Section S2 of the SI contains a self-contained derivation of the final expression for $S(\mathbf{I})$. What is important to note here, is that to spatially coarse-grain the dynamics we ”smear” the positions to the scale $\sigma$. This is equivalent to lowering the spatial resolution of the dynamics through a procedure that, in the framework of Renormalization Group theory, is commonly referred to as “position space regularization”.

In practice, we associate each configuration $Q_i\in {\mathscr {C}}$ with a finite region of the configuration space. We then derive the expression of the probability of a coarse-grained transition path (Fig. 2) by using a Feynmann path-integral. The explicit evaluation of this integral is quite lengthy and is detailed in section S2 of the SI. The final result is:

2 $$\begin{aligned} K_{{\text {cg}}}(Q_f, t|Q_i, t_i) =\int _{Q_i}^{Q_f}{\mathscr {D}}Q\,e^{-\frac{1}{2 m D_{\text {cg}}}\int _{t_i}^{t_f} d\tau \left( m\frac{\dot{Q}^2}{2} + V_{\mathrm {cg}}[Q(\tau )] \right) }. \end{aligned}$$

This is the probability in the coarse-grained dynamics to observe a transition between the regions $Q_f$ and $Q_i$ in a time $t-t_i$. $D_{\text {cg}}= \sigma ^2/(2 \Delta t)$ and $V_{\mathrm {cg}}(Q)$ are the diffusion coefficient and effective potential of the coarse-grained theory, respectively. The definition of $V_{\mathrm {cg}}(Q)$ and the numerical scheme we used to estimate it are also discussed in the SI.

*Path probability in the graph representation* Equation (2) leads to a closed expression for the coarse-grained effective path action entering Eq. (1) (for details, see section S5 in the SI):

3 $$\begin{aligned} S(\mathbf{I})= & {} \sum _{k} w_{i_{k+1} i_k}, \end{aligned}$$

where

4 $$\begin{aligned} w_{i j}\equiv & {} \frac{1}{2 m D_{\text {cg}}} \left( \frac{m (Q_{i}-Q_{j})^2}{2 \Delta t } + V_{{\text {cg}}}(Q_{i})\Delta t\right) . \end{aligned}$$

The exponential $e^{-w_{ij}}$ controls the probability of observing a transition between the regions centered around $Q_i$ and $Q_j$ of the intrinsic manifold, in an elementary (coarse-grained) time step $\Delta t$. We emphasize that, by definition of $D_{{\text {cg}}}$ and $V_{{\text {cg}}}$, the weights given in Eq. (4) are small numbers. As a result, probabilities of different paths on the graph are comparable. Further details about explicit evaluation of the weights $w_{ij}$ in a realistic application are reported in section S5 in the SI.

The present coarse-grained representation of the stochastic dynamics displays several analogies with Markov state modelling. A key difference is that, in our Renormalization Group inspired approach, the information about the kinetics is encoded in the renormalised effective potential, $V_{cg}(Q_i)$ and not in a stochastic transition matrix.

### Transition Path Sampling with a quantum annealer

Designing sampling algorithms exploiting quantum annealers has become a highly active research field^41–45^. We leverage on this development by integrating sampling via a quantum annealer into our classical–hybrid scheme, in order to generate realistic ensembles of coarse-grained transition pathways $\mathbf{I}$. To this end, we need to sample from a path distribution $\propto e^{-S(\mathbf{I})}$. In principle, conventional stochastic algorithms on a classical computer could serve this purpose. However, their computational cost grows very rapidly with the number of configurations in the data set ${\mathscr {C}}$. Ultimately, classical Markov chain Monte Carlo path sampling algorithms are typically limited by long auto-correlation times in the chain. As we will show below, quantum computers can overcome this limitation: in our approach each Monte Carlo step performed on D-Wave can generate a new uncorrelated transition path. Note that we do not require a fully fair sampling of the space of possible paths, which is one of the challenges in quantum-annealer based sampling^46–48^. Employing a suitable reweighting procedure, it is sufficient for our algorithm if the exploration of the accessible space is sufficiently broad.

*Quantum encoding of the transition path sampling problem* The first step to derive our path sampling algorithm consists in introducing a graph representation of the path probability density defined in Eq. (1). We identify each configuration in the data set ${\mathscr {C}}$ with a node in the graph and define the topology of the graph so to ensure that connected neighboring nodes represent configurations that are both structurally and kinetically close (in Section S3 of the SI, we provide further details on how we enforce this condition in the application to alanine dipeptide). The weights $w_{i j}$ of the edges in the graph are defined according to Eq. (4), thus ensuring that the sum of the weights along a given path $\mathbf{I}$ on the graph yields the path functional $S(\mathbf{I})$ entering Eq. (1).

The undirected graph representation enables us to map the sampling problem to a quantum annealing one. To this end, we introduce two sets of binary variables, $\Gamma ^{(1)}_i$ and $\Gamma ^{(2)}_{ij}$, where *i* and *j* run over the $\nu$ vertexes in the graph. If $\Gamma ^{(1)}_i=1$ ($\Gamma ^{(1)}_i=0$), then the *i*-th node is (is not) visited by the transition path on the graph (see red line in Fig. 2(b)). $\Gamma ^{(2)}_{ij}$ is always 0 if the *i* and *j* are not adjacent in the graph. If *i* and *j* are adjacent, then $\Gamma ^{(2)}_{ij}=1$ when the path contains the $i\rightarrow j$ or $j \rightarrow i$ transition. We are specifically interested in configurations of the binary variables in which the set of non-vanishing entries of $\Gamma ^{(1)}_i$ and $\Gamma ^{(2)}_{ij}$ form a topologically connected path, i.e., a continuous line starting from the given initial node and terminating in the chosen final node.

To sample path configurations according to $e^{-S(\mathbf{I})}$, let us consider the following classical Hamiltonian of the binary variables:

5 $$\begin{aligned} H = \alpha H_{{\text {C}}} + H_{{\text {T}}}. \end{aligned}$$

$H_{{\text {C}}}$ is the constraint Hamiltonian, a positive-definite function that is zero only if the entries of the binary variables satisfy the path topology, $H_{{\text {C}}}(\Gamma ^{(1)}, \Gamma ^{(2)})=0$. This condition can be fulfilled by choosing^49^

6 $$\begin{aligned} H_{{\text {C}}}= H_{{\text {s}}}+H_{{\text {t}}}+H_{\text {r}}, \end{aligned}$$

where

7 $$\begin{aligned} H_{{\text {s}}}=&-\left( \Gamma ^{(1)}_s\right) ^2+\left( \Gamma ^{(1)}_s-\sum _i\Gamma ^{(2)}_{s i}\right) ^2\,, \end{aligned}$$

8 $$\begin{aligned} H_{{\text {t}}}=&- \left( \Gamma ^{(1)}_t\right) ^2+\left( \Gamma ^{(1)}_t-\sum _i\Gamma ^{(2)}_{t i}\right) ^2\,, \end{aligned}$$

9 $$\begin{aligned} H_{{\text {r}}}=&\sum _{j\ne s,t} \left( 2\Gamma ^{(1)}_j-\sum _i\Gamma ^{(2)}_{j i}\right) ^2\,. \end{aligned}$$

In this formulation, $H_{\text {s}}$ and $H_{\text {t}}$ introduce the condition that the path should start from the initial node *s* and end in the final node *t*, while $H_{\text {r}}$ imposes the flux conservation at the remaining nodes.

$H_{\text {T}}= \sum _{ij} w_{ij}\,\Gamma ^{(2)}_{ij}$ is the so-called target function. By definition, $H_{\text {T}}$ yields the path action *S* whenever the configuration of the tensors $\Gamma ^{(1)}$ and $\Gamma ^{(2)}$ satisfy a path topology, that is, if $H_{\text {C}}( \Gamma ^{(1)},\Gamma ^{(2)})=0$ and $\mathbf{I}=\mathbf{I}(\Gamma ^{(1)}, \Gamma ^{(2)})$ is the corresponding path, then $H_{\text {T}}(\Gamma ^{(2)})=S(\mathbf{I})$. The parameter $\alpha$ in Eq. (5) controls the relative strength of the constraint Hamiltonian, $H_{\text {C}}$. For $\alpha \gg 1$, all binary variables’ configurations that violate the path topology correspond to very high excitations and are thus excluded from the sampling in the low-energy states that the quantum annealer performs.

Now, we are finally in a condition to tackle the problem of how to use a quantum annealer to sample path configurations with probability distribution $\propto e^{-S(\mathbf{I})}$. First, we use this machine to generate path-like binary variables’ configurations according to a probability distribution that has a finite overlap with $e^{-S(\mathbf{I})}$. Then, a classical machine accepts or rejects the proposal, thus restoring the correct path probability distribution $e^{-S(\mathbf{I})}$ (see Fig. 1).

To implement this scheme, we switch to a generalized Ising Model formulation of our classical Hamiltonian, by means of a change of variables: $\sigma _i^z=2 \Gamma ^{(1)}_i-1$, $\sigma _{ij}^z= 2 \Gamma ^{(2)}_{ij}-1$. Then, we promote the classical Eq. (5) to a quantum mechanical Hamiltonian ${{\hat{H}}}$, by substituting the classical Ising variables with Pauli *z* operators of a spin 1/2 algebra. Finally, the spin 1/2 states are encoded in the qubits of D-Wave.

In a standard quantum annealing process, the qubits are initialized in the ground-state of an easily solvable Hamiltonian^33–37^, in our case

10 $$\begin{aligned} {{\hat{H}}}_{\text {in}}= -h_{\mathrm {x}} \left( \sum _{i}{{\hat{\sigma }}}^x_i - \sum _{ij} {{\hat{\sigma }}}_{ij}^x\right) , \end{aligned}$$

where $h_{\mathrm {x}}$ is an arbitrary real constant. Then, the system is subjected to a time-dependent Hamiltonian

11 $$\begin{aligned} {{\hat{H}}}(t) = A(t)\,{{\hat{H}}}_{{\text {in}}} + B(t)\,{{\hat{H}}}, \end{aligned}$$

with scheduling functions *A*(*t*) and *B*(*t*). These are chosen such that initially $A(0) = 1$ and $B(0) = 0$, while at the end of the protocol, i.e., at $t=t_{{\text {sweep}}}$, one has $A(t_{{\text {sweep}}}) = 0$ and $B(t_{{\text {sweep}}}) = 1$. That is, the sweep starts with $H(0)=H_{\text {in}}$ and ends in $H(t_{{\text {sweep}}})= H$.

The spectrum of the quantum Hamiltonian ${{\hat{H}}}$ comprises all possible energy states of the classical Hamiltonian *H* defined in Eq. (5). Therefore, as long as $\alpha \gg 1$, the low-lying eigenstates of ${{\hat{H}}}$ represent path-like configurations $\mathbf{I}$ and their eigenvalues coincide with the path action $S(\mathbf{I})$. For a closed system, the adiabatic theorem implies that if the sweep is performed sufficiently slowly as compared to the minimal energy gap $\Delta E$, i.e., for $t_{{\text {sweep}}}\gg \hbar /\Delta E$, then the system remains in its instantaneous ground-state, thus reaching the lowest energy solution at the end of the sweeping process. In this ideal condition, the annealing process would systematically return the least action path ${\bar{\mathbf{I}}}$^25^. Since the path probability in Langevin dynamics is given by $\sim e^{-S(\mathbf{I})}$, the least action path ${\bar{\mathbf{I}}}$ corresponds to the most probable transition path.

In realistic conditions, the probability of landing onto the ground-state remains < 1, even in the limit of very long sweeping times. Fluctuations in the results can be due to a combination of different factors, including the thermal coupling of the machine to its environment and other dissipation effects, non-adiabatic corrections in the sweeping procedure, or even specific bias introduced by the hardware layout, such as, e.g., those inherent with the limited topological connectivity of the quantum annealing machine. Achieving unbiased results in the presence of such errors in realistic machines is an active area of research^46–48,50^. In our work, we chose a different approach and used the machine itself to compute the probability distribution of intercepting specific solutions in the low-lying sector of the spectrum, using a cumulant expansion approximation. Then, we have corrected for such distribution by introducing a reweighing term in the acceptance/rejection formula. We have added a paragraph after to clarify this point.

This is because the coupling of the quantum annealing device to its environment induces decoherence and thermal relaxation^51^. It has been suggested that this coupling can be exploited to sample classical Boltzmann distributions^52,53^. However, in practice, the sampling can only be performed at some rescaled temperature that is very difficult to estimate *a priori*^53^. The reason is that, if the coupling *A*(*t*) of the initial Hamiltonian $H_{{\text {in}}}$ decays sufficiently fast, the thermal relaxation time may grow longer than the sweeping time $t_{{{\text {sweep}}}}$, and the relaxation process freezes at some time $t_f<t_{{{\text {sweep}}}}$. In this case, the distribution of final energy states would be close to a modified Boltzmann distribution $e^{-B(t_f) S(\mathbf{I})}$, where $t_f$ is the freezing time. It should be emphasized, however, that the existing quantum annealing machines such as D-Wave, are very often employed in hybrid optimization schemes that combine classical and quantum annealing. In this case, we do not expect the path probability should correspond to a Boltzmann distribution. In addition to fluctuations due to coupling to an environment, we exploit quantum fluctuations in the final state due to non-adiabaticity generated by relatively rapid sweeps. In the following, we only assume that there exists a regime of sweeping times for which the distribution of the paths generated by multiple hybrid energy minimization has a finite overlap with $e^{-S(\mathbf{I})}$.

A classical computer controlling a Metropolis scheme can exploit this overlap to yield the correct sampling of $e^{-S(\mathbf{I})}$ (Fig. 1). In general, this can be achieved by imposing the detailed balance condition $e^{-S(\mathbf{I})} T( \mathbf{I}'|\mathbf{I}) = e^{-S(\mathbf{I}')} T(\mathbf{I}|\mathbf{I}')$, where $T(\mathbf{I}'| \mathbf{I})$ is the transition probability from the path $\mathbf{I}$ to the path $\mathbf{I}'$ in the underlying stochastic process. We choose to generalize this dynamics to enable also the sweeping time $t_{{{\text {sweep}}}}$ to vary along the Markov chain. We do so to ensure that D-Wave is mostly performing sweeps with a duration $t_{{{\text {sweep}}}}\sim t_0$, where $t_0$ is a tunable parameter representing a reasonable compromise between accuracy (slow sweeping) and efficiency (low consumption of quantum computing time). Upon enlarging the configuration space of the Monte Carlo dynamics to include $t_{{{\text {sweep}}}}$, the new detailed balance condition reads $\rho (\mathbf{I}, t_{{{\text {sweep}}}})\, T(t_{{{\text {sweep}}}}', \mathbf{I}'|t_{{{\text {sweep}}}}, \mathbf{I}) = \rho (\mathbf{I}', t_{{{\text {sweep}}}}')\,T(t_{{{\text {sweep}}}}, \mathbf{I}|(t_{{{\text {sweep}}}}', \mathbf{I}')$, where $\rho (\mathbf{I}, t_{{{\text {sweep}}}})$ is the new equilibrium distribution. Our Monte Carlo dynamics must be defined in such a way to ensure that the equilibrium distribution is

12 $$\begin{aligned} \rho (t_{{{\text {sweep}}}}, \mathbf{I}) = p_0(t_{{{\text {sweep}}}})\times e^{-S(\mathbf{I})}, \end{aligned}$$

where $p_0(t_{{{\text {sweep}}}})$ is some arbitrary equilibrium distribution of the sweeping time, centered around $t_0$. Following the standard procedure to obtain the Metropolis acceptance/rejection criterium, we write the transition probability as a product of a trial move probability $\tau (\mathbf{I}', t_{{{\text {sweep}}}}'|\mathbf{I}, t_{{{\text {sweep}}}})$ and a corresponding acceptance probability $a(\mathbf{I}', t_{{{\text {sweep}}}}'|\mathbf{I}, t_{{{\text {sweep}}}})$. Since the sweeping time is allowed to vary along the chain, we factorize the trial move probability as

13 $$\begin{aligned} \tau (\mathbf{I}', t_{{{\text {sweep}}}}'| \mathbf{I},t_{{{\text {sweep}}}}) = P(t_{{{\text {sweep}}}}'|t_{{{\text {sweep}}}}) \,P(\mathbf{I}'|t_{{{\text {sweep}}}}'), \end{aligned}$$

where $P(t_{{{\text {sweep}}}}'| t_{{{\text {sweep}}}})$ is the probability for the sweeping time to go from $t_{{{\text {sweep}}}}$ to $t_{{{\text {sweep}}}}'$ in a Monte Carlo step, while $P(\mathbf{I}|t_{{{\text {sweep}}}})$ is the probability that a quantum annealing calculation lasting a time $t_{{{\text {sweep}}}}$ yields the path $\mathbf{I}$. Combining all terms together, we obtain the following Metropolis acceptance rule:

14 $$\begin{aligned} {\text {min}}\left[ 1, \frac{p_0(t_{{{\text {sweep}}}}')\,P(t_{{{\text {sweep}}}}| t_{{{\text {sweep}}}}')}{p_0(t_{{{\text {sweep}}}})\,P(t_{{{\text {sweep}}}}'| t_{{{\text {sweep}}}})} \frac{P(\mathbf{I}|t_{{{\text {sweep}}}})}{P(\mathbf{I}'|t_{{{\text {sweep}}}}')} \frac{e^{-S(\mathbf{I}')}}{e^{-S(\mathbf{I})}}\right] \end{aligned}$$

In particular, in our simulations we chose to update $t_{{{\text {sweep}}}}$ according to a Brownian dynamics with a harmonic drift term:

15 $$\begin{aligned} t_{{\text {sweep}}}^{i+1} = t_{{\text {sweep}}}^i -\delta t k (t_{{{\text {sweep}}}}-t_0) + \sqrt{2 \delta t} \xi ^i, \end{aligned}$$

where $\xi _i$ is a Gaussian distributed random variable of null mean and unitary variance and $\delta t$ is an incremental sweeping time change.

The conditional probability $P(\mathbf{I}|t_{{{\text {sweep}}}})$ in Eq. (14) depends on the details of the quantum annealing machine and of the specific optimization algorithm. In general, computing $P(\mathbf{I}| t_{{{\text {sweep}}}})$ from a theoretical model of the annealing process can be very challenging. We overcome this problem and show how to estimate $P(\mathbf{I}|t_{{{\text {sweep}}}})$ by performing a moderate number of annealing processes, for each value of $t_{{\text {sweep}}}$. The spectrum of the target quantum Hamiltonian ${{\hat{H}}}$ is expected to be non-degenerate, since the weights in the graph $w_{ij}$ are in general all different. In addition, for large values of the parameter $\alpha$ in Eq. (5), all low-lying states satisfy the constraints set by $H_{\text {C}}$, and thus correspond to path-like configurations $\mathbf{I}$. Therefore, each low-lying eigenvalue *E* of the quantum Hamiltonian ${{\hat{H}}}$ corresponds to the action of a single path, $E=S(\mathbf{I})$. Then, $P(\mathbf{I}|t_{{{\text {sweep}}}})$ can be directly inferred from a frequency histogram of the energies *E* obtained at the end of multiple annealing processes performed at fixed $t_{{{\text {sweep}}}}$, i.e., $P(\mathbf{I}|t_{{{\text {sweep}}}})=P(E| t_{{{\text {sweep}}}})$. To minimize the consumption of quantum computing time, we can estimate $P(E| t_{{{\text {sweep}}}})$ by the lowest-order cumulant expansion as

16 $$\begin{aligned} P(\mathbf{I}|t_{{{\text {sweep}}}}) \simeq P(E|t_{{{\text {sweep}}}}) \simeq \frac{1}{\sqrt{2 \pi } \Delta } e^{-\frac{(E(\mathbf{I})-{\overline{E}})^2}{2 \Delta ^2}}, \end{aligned}$$

where ${\overline{E}}$ and $\Delta$, respectively, are the average and the standard deviation of the energy obtained by many annealing processes at fixed sweeping time $t_{{{\text {sweep}}}}$. In principle, the estimated distribution can be improved systematically by including higher orders in the cumulant expansion, which may in particular become important in the presence of long tails. For our benchmark purposes, we find (16) to be sufficient.

## Application to a molecular benchmark system

To illustrate our hybrid classical/quantum Monte Carlo scheme sketched in Fig. 1, we apply it to simulate the $C5\rightarrow \alpha _R$ transition of alanine dipeptide. We have chosen this standard benchmark system, as it is sufficiently small to enable us to carry out the quantum computing calculations on existing D-Wave machines. This serves to illustrate all the relevant steps, which—thanks to the generality of our approach—can be applied to molecular systems of increased complexity in step with increasing qubit numbers in future generations of quantum annealers.

First, we use iMapD and our effective Langevin theory to construct the graph representation of the dynamics on the intrinsic manifold of this peptide (details on the implementation of iMapD and the calculation of the weights $w_{ij}$ for this molecular system are provided in section and S5 of the SI). The results are shown in the Ramachandran plot reported in Fig. S2–S4 of the SI. The contour lines in the background represent the free energy surface, calculated from a frequency histogram of $1\,\mu$s of equilibrium MD at $T=300\,$K, generated using OpenMM^54^, in the AMBER99SB force field with explicit TIP3P water^55^. The spatial resolution of our effective theory is determined by the number of configurations $\nu$ we keep to generate a sparse graph. With this choice, the average RMSD distance between neighbouring configurations in our network is $\delta _{\mathrm {RMSD}}\simeq 0.5$ $\text{\AA}$. Then, $\sigma \simeq \delta _{\mathrm {RMSD}} \sqrt{N_a}$, where $N_a=22$ is the number of atoms in our molecule.

To implement our hybrid classical/quantum Monte Carlo scheme, we encode the quantum Hamiltonian ${{\hat{H}}}$ defined by the graph using the Ocean suite, operating on the D-Wave quantum annealer. Encoding our system on D-Wave requires 578 qbits, given by the sum of the number of nodes and edges of our network. To generate trial paths, we rely on the hybrid solver available on Leap, which combines quantum annealing with classical simulating annealing. In this case, $t_{{{\text {sweep}}}}$ is identified with the total quantum and classical computing time employed by the solver. We estimate the resulting conditional probability $P(\mathbf{I}|t_{{{\text {sweep}}}})$ entering Eq. (14) by means of a direct calculation on D-Wave (Table S1 in SI), using Eq. (16). In Fig. S8 in the SI, we report the average value of the energy ${\overline{E}}$ and its standard deviation $\Delta$, entering Eq. (16).

We initiated three independent Markov chains from arbitrary paths generated by a quantum annealing process at $t_{{{\text {sweep}}}}=180\,\hbox {s}$, $30\,\hbox {s}$, and $240\,\hbox {s}$, corresponding to about $8.6\,\hbox {s}$, $1.4\,\hbox {s}$, and $11.4\,\hbox {s}$ of quantum annealing time, respectively (details on how we determine the initial and final nodes are outlined in Section S3 of the SI). We evolved $t_{{{\text {sweep}}}}$ according to Eq. (15) with $k=2\times 10^{-4}\,\hbox {s}$ $^{-1}$ and $t_0=150$s and then accepted or rejected the new paths according to Eq. (14).

We recall that the $\alpha$ parameter determining the relative strength of the constraint and target Hamiltonian must be chosen in order to balance between two general requirements: (i) it needs to be as large as necessary to energetically separate configurations with false topology reliably from the correct low-energy manifold and (ii) it needs to be as small as possible, since the maximal achievable energy scale is limited by the hardware; thus, a larger value of $\alpha$ would in fact correspond to a decrease of all the other energy scales and hence to a worse energetic resolution of the configurations with correct topology. In this application, we heuristically set $\alpha =\sum _{ij}w_{ij}$, which is compliant with both the aforementioned requests. With this choice, on average, over 60% of the annealing sweeps led to configurations of binary variables $\Gamma ^{(1)}$ and $\Gamma ^{(2)}$ with a correct path topology (Table S2 in SI), thus providing viable trial transition paths.

**Figure 3:**
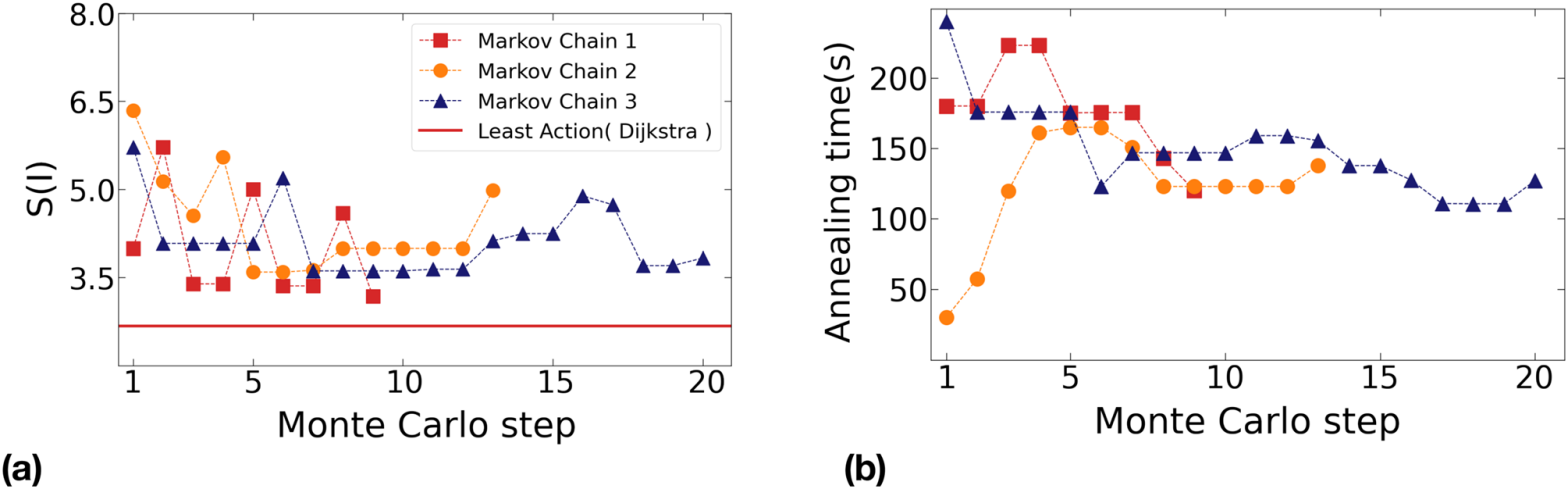
(a) Evolution of the path action $S(\mathbf{I})$ and (b) annealing time $t_{{{\text {sweep}}}}$ along the Monte Carlo paths generated using the hybrid classical/quantum annealing implemented on D-Wave.

In Fig. 3 we show the change in path action *S* (left panel) and the hybrid minimization time $t_{{{\text {sweep}}}}$ (right panel), along our three Markov chains. As these results show, the Monte Carlo algorithm occasionally accepts trial moves with a higher action. They also show that longer annealing times do not always yield paths with lower actions. This is expected, since the $P(E|t_{{{\text {sweep}}}})$ distributions have significant overlap, as it can be inferred from Fig. S8 in the SI.

**Figure 4:**
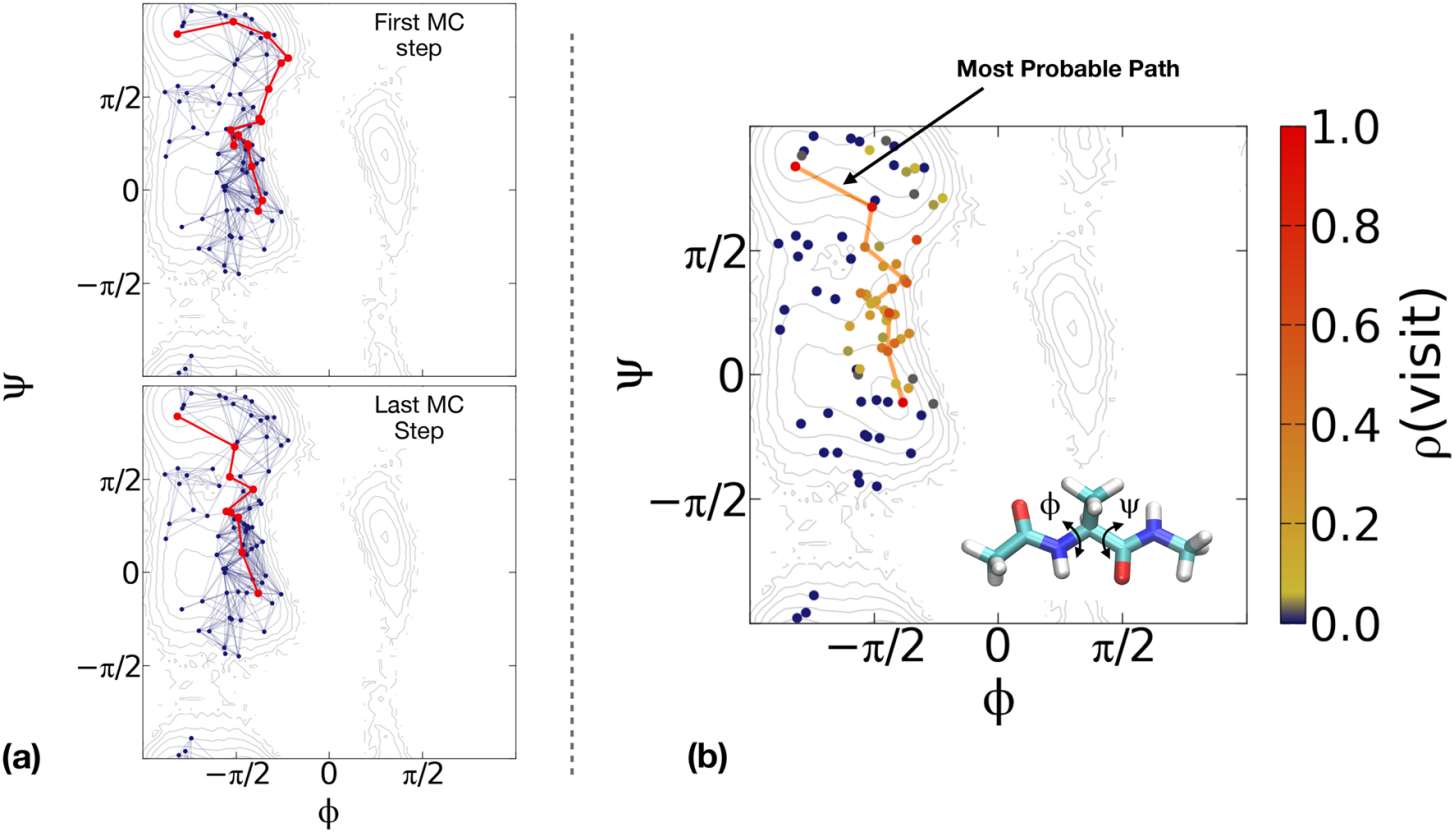
(**a**) Transition pathways for the $C5\rightarrow \alpha _R$ transition of alanine dipeptide obtained from our Monte Carlo scheme. The red line denotes the first (top) and last (bottom) trajectory in a Markov chain. The points in the Ramachandran plots are obtained from projecting the configurations generated with iMapD. In the background is the free energy surface calculated from 1 μs of plain MD. (**b**) Transition path density on the Ramachandran plane, evaluated for the ensemble of trajectories calculated with our Monte Carlo scheme. The solid orange line is the most probable path, obtained using the Dijkstra algorithm^59^ on a classical computer.

The transition paths generated by our scheme are consistent with the free energy landscape produced by equilibrium MD. Figure 4a shows the first and last accepted transition paths of one of the generated Markov chains. Both paths correctly connect the two meta-stable states, navigate the low-free energy regions of the surface, and cross the barrier at its lowest point.

The transition paths explore a region around the most probable path^56–58^, which in Fig. 4b is shown based on calculations on a classical computer using the Dijkstra algorithm^59^. Figure 4b also reports how often the sampled transition paths pass the nodes of the network, i.e., the statistical weight of the corresponding configuration in the transition path ensemble. All transition paths go through the transition state. However, due to the presence of fluctuations, a finite probability is obtained also at configurations with relatively high free energy. The deterministic Dijkstra algorithm can only detect the global minimum of the functional $S(\mathbf{I})$. In contrast, our TPS algorithm accounts for fluctuations that lead to the full transition path ensemble.

**Figure 5:**
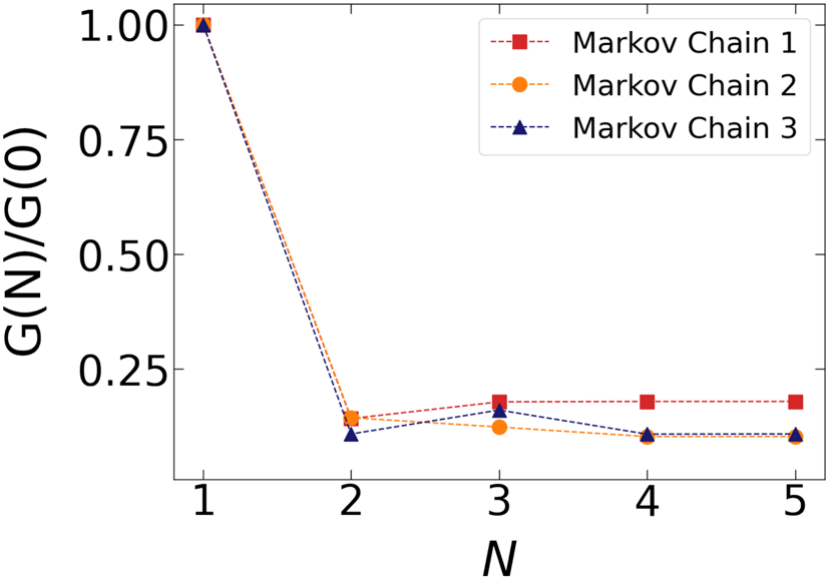
The ratio of auto-correlation function *G*(*N*)/*G*(0) (see Eq. (S29) in SI) plotted as a function of Monte Carlo steps *N* for three independent Markov chains.

The main strength of our hybrid classical/quantum scheme is that it allows us to efficiently obtain independent transition paths. At each adiabatic cycle, the quantum machine is reset to a quantum state in which all the spins are aligned along the ${{\hat{x}}}$ direction. Consequently, each qubit has initially 50% probability to be in configuration + 1/2 and $-1/2$. Since the only source of correlation is introduced by the Markovian stochastic evolution of the minimization time $t_{{{\text {sweep}}}}$ (Eq. (15)), we expect each minimization procedure to yield a completely independent path (one-shot generation). To quantify the degree of correlation in the ensemble of trajectories sampled in three Markov chains, we consider the auto-correlation function *G*(*N*) defined in the Section S6 of the SI, where *N* denotes distance in the Monte Carlo chain. In Fig. 5 we plot the behaviour of *G*(*N*) (evaluated relatively to its initial value *G*(0) ) for each independent Markov chain (see also discussion in Section S6 of the SI). These results clearly indicate that the correlation of the generated trajectories is suppressed after just a single Monte Carlo step.

## Conclusions

In this work, we have established a novel computational framework to sample the transition path ensemble of molecular conformational transitions, which integrates a ML driven exploration with a hybrid Monte Carlo scheme that exploits the potential of QC. We have used the iMapD algorithm^32^ to achieve an uncharted exploration of the molecular intrinsic manifold, without introducing any choice of CV, nor biasing force. These data enabled us to build a coarse-grained representation of the dynamics directly on the intrinsic manifold. To construct this low-resolution theory, we adapted regularization and renormalization methods that were originally developed within the context of high energy physics^40^, and which may also be useful in other applications in soft-condensed matter and biophysics^60,61^. We then encoded the path sampling problem in a form that enabled us to use a D-Wave quantum annealer to generate uncorrelated trial transition paths, thus enhancing the exploration of the transition path ensemble. Finally, by using the Metropolis criterion in Eq. (14), we made sure to account for each trajectory with its correct statistical weight in the transition path ensemble.

The algorithm we presented here is designed to sample the full transition path ensemble. This achievement represents a significant advancement with respect to previous attempts to compute the most probable transition paths on a quantum computer^25^. The transition path ensemble is often heterogeneous, displaying several alternative transition channels, corresponding to alternative molecular mechanisms. Even though in the proof-of-concept we discussed here we restricted our sampling to 23 transition paths, in general the number of trajectories is only limited by the available computational resources.

While significant effort has been made towards designing quantum algorithms for quantum many-body problems^19–24,62–66^, only a few applications of quantum computing to classical molecular sampling problems have been reported to date^25,26,67–69^. Most of these attempts assume a simplified molecular representation, among which lattice discretization^26,67,68^. Unlike the method developed in^25^, which was designed to return only the most probable path, here we sample the full transition path ensemble. In addition, we do not introduce any unphysical biasing force nor a choice of CVs to accelerate the exploration of configuration space. Finally, to the best of our knowledge, the present calculation represents the first successful application of a quantum computing machine to characterize a molecular transition using a state-of-the-art atomistic force field.

With the present quantum encoding, the size of the molecular systems that can currently be investigated is limited by the relatively small number of qubits that are available on the existing quantum annealing machines. The characterization of transitions with a comparable level of spatio-temporal resolution of much larger molecules (for example, the folding of a small protein) typically requires to generate at least 10^3^ −  10^4^ points on the intrinsic manifold^25^. In this case, implementing our scheme on a quantum computer would require a number of qubits more than one order of magnitude larger than that of the most powerful existing quantum annealing device. However, if the size of quantum computing hardware continues to grow in size and performance according to the present exponential rate^27–29^, we may hope this threshold to be reached within the foreseeable future. Due to the suppression of autocorrelation time, we expect that, for sufficiently large networks, our hybrid scheme may ultimately have an edge over classical stochastic methods.

In the future, heterogeneous platforms for high-performance computing might emerge that integrate CPUs and GPUs with quantum and machine learning cores. These new machines will require scientific software able to operate across the different parts, fully taking advantage of their strengths. Heterogeneous algorithms like the one we presented here will thus become increasingly important, with great potential for the computational molecular sciences.

## Supplementary Information

Supplementary Information.

## Data Availability

The datasets used and/or analysed during the current study are available from the corresponding author on reasonable request.
