# Supplementary material for: Sampling rare conformational transitions with a quantum computer
Source: Sci Rep. 2022 Sep 29;12:16336. doi: 10.1038/s41598-022-20032-x (PMC9522734; doi:10.1038/s41598-022-20032-x)
Supplement: Supplementary file 1 — Supplementary Information. [file 41598_2022_20032_MOESM1_ESM.pdf]

Sampling Rare Conformational Transitions with a Quantum Computer: Supplementary Material

Danial Ghamari[1,2], Philipp Hauke[3], Roberto Covino[4,*], and Pietro Faccioli[1,2,*]

[1]University of Trento, Department of Physics, Via Sommarive 14, Trento, I-38123, Italy
[2]INFN-TIFPA, Via Sommarive 14, Trento, I-38123, Italy
[3]University of Trento, INO-CNR BEC Center & Department of Physics, Via Sommarive 14, Trento, I-38123, Italy
[4]Frankfurt Institute for Advanced Studies, Ruth-Moufang-Straße 1, Frankfurt am Main, D-60438, Germany
[*]covino@fias.uni-frankfurt.de
[*]pietro.faccioli@unitn.it

ABSTRACT

In this Supplementary Information, we provide details about aspects of the framework we laid out in the main text. In particular, in section S1, S3, and S4 we provide details on the uncharted exploration of underlying manifold using intrinsic map dynamics (iMapD)[1] and its application to alanine dipeptide. In section S2, we provide a detailed and self-contained derivation of the spatially coarse-grained dynamics of a diffusive process in a path-integral formalism. In section S5 we explain the details of the graph weights w_{ij} calculationthe in the application of our algorithm to alanine dipeptide. Finally, in section S6, we provide the definition of the auto-correlation function $G(N)$ discussed in the main text.

S1: Details of the the intrinsic manifold exploration

We start by performing short unbiased sampling in each meta-stable state. Here, we assume that the interesting meta-stable states are known and that representative structures are available, as it is often the case. Each local sampling returns a set of M configurations $\mathscr{C}_{\text{ini}} = \{Q_1, \ldots, Q_M\}$, with $\dim(Q_i) = 3N_a$. We identify the boundary of $\mathscr{C}_{\text{ini}}$ in the low-dimensional intrinsic manifold defined by diffusion maps[2]. For each couple of configurations Q_i and Q_j, we calculate the pairwise root-mean-square-deviation (RMSD) on all non-hydrogen atoms after removing global translations and rotations, and use it as a metric, $d_{ij} = \min \text{RMSD}(Q_i, Q_j)$. We then construct a transition matrix by calculating

$$P_{ij} = C\, e^{-d_{ij}^2/\varepsilon^2}\,, \tag{S.1}$$

where ε is a distance threshold that defines a notion of neighbourhood in the configuration space, here defined as $\varepsilon = \sigma(d_{ij}) - \Delta\sigma(d_{ij})$, where σ is the average of the pairwise distances, and $\Delta\sigma$ its standard deviation. We use here a Gaussian kernel, which is a popular choice, but other choices are possible[2]. The constant C provides a normalization such that the sum along each row of P_{ij} is one. Solving the eigenvalue problem for P_{ij}, we obtain eigenvalues λ_k and eigenvectors ψ_k, where $k = 0, \ldots, M-1$. A projection of the high-dimensional data $\mathscr{C}_{\text{ini}}$ onto a $n \ll N$ low-dimensional embedding $\mathbf{z} = \{z_1, ..., z_M\}$ is established via the components of n dominant eigenvectors[2], i.e., each Q_i gets mapped into $z_i = \{\psi_1(i), \ldots \psi_n(i)\}$, where $\psi_k(i)$ is the i-th component of eigenvector ψ_k. We identify points on the boundary of $\mathscr{C}_{\text{ini}}$, the set of which we denote $\mathscr{C}^B_{\text{ini}}$, as those defining a convex hull containing the low-dimensional embedding $\mathbf{z}$.

We then generate new configurations in the unexplored regions by "shooting" beyond the boundary of the known configurations space. For each point Q^B_i in $\mathscr{C}^B_{\text{ini}}$, we first identify the set of nearest neighbours within a given distance $\delta = \sigma(d_{ij}) - \Delta\sigma(d_{ij})$. For each such set, we calculate the projections of all points in the principal component analysis (PCA) representation, i.e., $q_i = Q_i\mathbf{V}$, where lower case indicates a sample projected on the principal components, upper case in the configuration space, and $\mathbf{V}$ is a matrix containing the PCA loadings[1]. For every point on the boundary and its neighborhood, we generate a new unexplored configuration beyond the boundary with

$$q_{\text{new}} = q_{\text{b}} - q_c + c\,\frac{q_{\text{b}} - q_{\text{c}}}{|q_{\text{b}} - q_{\text{c}}|}\,. \tag{S.2}$$

Here, q_{new}, q_{b}, and q_{c} are respectively coordinates in the principal component projection for the new data point, boundary point, and the center of mass of the neighboring set without including the boundary point. The constant $c > 0$, which is adjusted heuristically, controls how far away beyond the boundary we generate new configurations. Finally, we retrieve the Cartesian coordinates of the new configuration with $Q_{\text{new}} = q_{\text{new}}\mathbf{V}^T + Q_c$.

The exploration proceeds iterating between two steps: generating new configurations beyond the boundary and starting short rounds of unbiased sampling from these configurations. At every step i, we generate one new configuration for every point on the boundary of the data set of all configurations sampled up to step $i-1$. We then merge together all configurations sampled up to step i and identify a new boundary, which is then used in the $i+1$-th iteration.

S2: Detailed derivation of a renormalized coarse-grained dynamics

We assume the system obeys a Langevin dynamics in the overdamped limit,

$$\dot{Q}(t) = -\frac{1}{m\gamma}\nabla U(Q) + \eta(t)\,, \tag{S.3}$$

where Q is a point in the molecular configuration space, m and γ are the atomic mass and viscosity (assumed for simplicity to be uniform and isotropic), $U(Q)$ is the potential energy, and $\eta(t)$ is a white noise of null average obeying the fluctuation-dissipation relationship

$$\langle \eta(t)\cdot\eta(0)\rangle = 6DN_a\delta(t), \tag{S.4}$$

where N_a is the number of atoms and $D = k_\mathrm{B}T/m\gamma$ is the diffusion coefficient. The probability of observing a system described by Eq. (S.3) in a given configuration at a given time obeys the Fokker–Planck equation:

$$\frac{\partial}{\partial t}\quad P(Q,t) = D\left(\nabla^2 + \tfrac{1}{k_\mathrm{B}T}\nabla U(Q)\cdot\nabla + \tfrac{1}{k_\mathrm{B}T}\nabla^2 U(Q)\right)P(Q,t)\,. \tag{S.5}$$

The solution of this equation can be conveniently expressed in path-integral form[3–5],

$$P(Q_f,t_f|Q_i,t_i) = \int_{Q_i}^{Q_f}\mathscr{D}Q\; e^{-S_\mathrm{OM}[Q]}, \tag{S.6}$$

where $S_\mathrm{OM}[Q] = \frac{1}{4D}\int_{t_i}^{t_f} d\tau \left(\dot{Q} + \frac{D}{k_\mathrm{B}T}\nabla U[Q(\tau)]\right)^2$ is the so-called Onsager–Machlup functional[6].

To build a coarse-grained representation of the molecular dynamics with a low spatio-temporal resolution, it is convenient to formulate the stochastic dynamics defined by the Fokker–Planck Equation (S.5) using the mathematical formalism of quantum mechanics. Substituting $P(Q,t) = e^{-\frac{U(Q)}{2k_\mathrm{B}T}}\Psi(Q,t)$ into the Fokker–Planck equation, we obtain the imaginary time Schrödinger equation,

$$-\hbar_\mathrm{eff}\partial_t\Psi(Q,t) = \hat{H}_\mathrm{eff}\Psi(Q,t), \tag{S.7}$$

where we have introduced an effective "Dirac's constan" $\hbar_\mathrm{eff} = \frac{2k_\mathrm{B}T}{\gamma}$ that controls the amount of thermal fluctuations in the system and $\hat{H}_\mathrm{eff} = -\frac{\hbar^2_\mathrm{eff}}{2m}\nabla + V_\mathrm{eff}(Q)$ is an effective quantum Hamiltonian, with an effective potential

$$V_\mathrm{eff}(Q) = \frac{1}{2m\gamma^2}\left[|\nabla U(Q)|^2 - \hbar_\mathrm{eff}\gamma\nabla^2 U(Q)\right]. \tag{S.8}$$

Similarly, equation (S.6) can be re-written as an imaginary-time quantum Feynman propagator[7,8]

$$P(Q_f,t|Q_i,t_i) = e^{-\frac{1}{2k_\mathrm{B}T}\left(U(Q_f)-U(Q_i)\right)}\,K(Q_f,t|Q_i,t_i), \tag{S.9}$$

$$K(Q_f,t|Q_i,t_i) = \langle Q_f|e^{-\frac{1}{\hbar_\mathrm{eff}}\hat{H}_\mathrm{eff}(t-t_i)}|Q_i\rangle = \int_{Q_i}^{Q_f}\mathscr{D}Q\; e^{-\frac{1}{\hbar_\mathrm{eff}}\int_{t_i}^{t_f}d\tau\left(\frac{\dot{Q}^2}{2}m + V_\mathrm{eff}[Q(\tau)]\right)}. \tag{S.10}$$

We note that the pre-factor $e^{-\frac{1}{2k_BT}\left(U(Q_f)-U(Q_i)\right)}$ in Eq. (S.9) does not affect the relative statistical weight of transition paths sharing identical boundary conditions.

The description of stochastic dynamics provides a natural framework to devise a systematic coarse-grained scheme, e.g. by applying renormalization group methods. In particular, we are interested in developing a low spatio-temporal resolution approximation of our original microscopic stochastic dynamics, characterized by a spatial resolution σ on the order of the typical distance between nearest neighbour configurations generated by iMapD. The time resolution Δt of this coarse-grained theory represents the shortest time scale, thus must be much smaller than the typical time it takes the system to diffuse across neighboring configurations in $\mathscr{C}$.

To lower the spatial resolution of the original stochastic theoy, we define a set of so-called coarse-grained states in the Hilbert space of the effective quantum mechanical theory:

$$|Q\} \equiv \int d^{3N_a}Q' \; \phi(Q'-Q)|Q'\rangle \tag{S.11}$$

In this expression, $\phi(Q)$ denotes a fast-decaying smooth smearing function centered around 0, such that $|\phi(Q)| \xrightarrow{|Q|\gg\sigma} 0$. We determine $\phi(Q)$ by requiring the orthonormality condition

$$\{Q|Q'\} = \delta_\sigma(Q-Q'), \tag{S.12}$$

where $\delta_\sigma(Q)$ is a coarse-grained representation of Dirac's delta function, smeared to the resolution scale σ. In particular, choosing a Gaussian smearing

$$\delta_\sigma(Q) = \frac{e^{-\frac{Q^2}{2\sigma^2}}}{(\sqrt{2\pi}\sigma)^{3N_a}} \tag{S.13}$$

yields $\phi(Q) = \frac{1}{(\sqrt{\pi}\sigma)^{3N_a}} e^{-\frac{Q^2}{\sigma^2}}$.

Smearing the position eigen-states to the scale σ using Eq. (S.11) is equivalent to lowering the spatial resolution by cutting off large momentum states, with $p \gtrsim \hbar_{\rm eff}/\sigma$. In the framework of Renormalization Group theory, this procedure is commonly referred to as "regularization". In practice, we associate each configuration $Q_i \in \mathscr{C}$ with a finite region of the configuration space, identify it with a coarse-grained state $|Q_i\}$.

To assign a finite probability to coarse-grained paths (Fig.2 in the main text), we need a path integral expression for the Feynman propagator defined on the coarse-grained states, i.e. $K_{\rm cg}(Q_f,t|Q_i) \equiv \{Q_f|e^{-\frac{1}{\hbar_{\rm eff}}\hat{H}_{\rm eff}t}|Q_i\}$. The explicit evaluation of this propagator is quite lengthy and is detailed in the following sub-section. The final result is:

$$K_{\rm cg}(Q_f,t|Q_i,t_i) = \int_{Q_i}^{Q_f} \mathscr{D}Q \; e^{-\frac{1}{2mD_{\rm cg}}\int_{t_i}^{t_f} d\tau\left(m\frac{\dot{Q}^2}{2}+V_{\rm cg}[Q(\tau)]\right)}, \tag{S.14}$$

where $D_{\rm cg} = \sigma^2/(2\Delta t)$ and $V_{\rm cg}(Q)$ are the diffusion coefficient and effective potential of the coarse-grained theory, respectively. The numerical scheme we used to estimate it are also discussed in section S5.

Feynman propagator in the coarse-grained theory

In this section, we derive our coarse-grained expression for the Feynman propagator of our coarse-grained theory describing the stochastic dynamics on the intrinsic manifold. In practice, this corresponds to evaluating the matrix element of the evolution operator as a path integral performed over the coarse-grained states $|Q\}$. In our specific implementation, the configurations specifying the center of the coarse-grained states lie on the intrinsic manifold and belong to the data-set generated with iMapD, i.e. $Q \in \mathscr{C}$.

The Feynman propagator reads

$$K_{\rm cg}(Q_f,t|Q_i) = \{Q_f|\hat{U}(t)|Q_i\}, \tag{S.15}$$

where $\hat{U}(t) = e^{-\frac{t}{\hbar_{\rm eff}}\hat{H}_{eff}}$ is the time evolution operator. Following the standard derivation of the Feynman's path integral representation of the propagator, we begin by carrying out a Trotter decomposition:

$$K_{\rm cg}(Q_f,t|Q_i) = \int {\rm d}Q_0 \ldots dQ_{N_t} \prod_{n=0}^{N_t-1} \left[\{Q_{n+1}|\hat{U}(\Delta t)|Q_n\}\right] \delta_R(Q_{N_t}-Q_f)\,\delta_\sigma(Q_0-Q_i), \tag{S.16}$$

where, Δt is a time scale of the order of the time resolution of the coarse-grained theory.

Let us focus on the elementary propagator $\{Q_{n+1}|\hat{U}(\Delta t)|Q_n\}$. As usual, by choosing a sufficiently small discretization time, we can ignore commutations between kinetic and potential operators and factorize the evolution operator

$$\hat{U}(\Delta t) \;\; \simeq \;\; e^{-\frac{1}{\hbar_{\rm eff}}\hat{T}_{\rm eff}\Delta t} e^{-\frac{1}{\hbar_{\rm eff}}\hat{V}_{\rm eff}\Delta t}, \tag{S.17}$$

where corrections are of higher order in Δt. Using Eq. (S.11), we obtain

$$\{Q_{n+1}|U(\Delta t)|Q_n\} = \int dz \int dz' \int \frac{d^{3N_a}P}{(2\pi\hbar_{\rm eff})^{3N_a}} e^{-\frac{\Delta t P^2}{2m\hbar_{\rm eff}}} e^{\frac{i}{\hbar_{\rm eff}}P\cdot(z'-z)} \phi(Q_{n+1}-z')\phi(Q_n-z) e^{-\frac{\Delta t}{\hbar_{\rm eff}}V_{\rm eff}(z)}. \tag{S.18}$$

By using the definition of the smearing function $\phi(Q)$ and performing the momentum integral, this expression is approximated as

$$\{Q_{n+1}|U(\Delta t)|Q_n\} \simeq \mathscr{N}\int dz\int dz' e^{-\frac{m(z-z')^2}{2\hbar_{\text{eff}}\Delta t}} e^{-\frac{1}{\sigma^2}((Q_{n+1}-z')^2+(Q_n-z)^2)} e^{-\frac{dt}{2\hbar_{\text{eff}}}V_{\text{eff}}(z)}, \tag{S.19}$$

where $\mathscr{N}$ is an irrelevant normalization constant.

We now recall that Δt is by definition the smallest time scale of our effective theory. Then, the Gaussian factor $e^{-\frac{m(z-z')^2}{2\hbar_{\text{eff}}\Delta t}}$ in Eq. (S.19) remains finite only when the distance between z and z' lies within the spatial resolution of the coarse-grained theory, σ, and it can be effectively regarded as a Dirac delta-function. After carrying out the integral in dz', we obtain

$$\{Q_{n+1}|U(\Delta t)|Q_n\} \simeq \mathscr{N} e^{-\frac{1}{2\sigma^2}(Q_{n+1}-Q_n)^2}\int dz \frac{e^{-\frac{2}{\sigma^2}\left(z-\frac{Q_{n+1}+Q_n}{2}\right)^2}}{(\sqrt{2\pi}(\sigma/2))^{6N_a}} e^{-\frac{\Delta t}{\hbar_{\text{eff}}}V_{\text{eff}}(z)}. \tag{S.20}$$

We can cast this expression in a more familiar form by introducing a smeared effective potential $V_{\text{s}}(Q)$ defined as follows:

$$e^{-\frac{\Delta t}{\hbar_{\text{eff}}}V_s(x)} \equiv \int dz \frac{e^{-\frac{2}{\sigma^2}(z-x)^2}}{(\sqrt{2\pi}(\sigma/2))^{6N_a}} e^{-\frac{\Delta t}{\hbar_{\text{eff}}}V_{\text{eff}}(z)}. \tag{S.21}$$

We emphasize that the average in the right-hand side is dominated by the configurations with the lowest effective potential. From the definition of V_{eff}, it follows that these are configurations near mechanical equilibrium points, where the modulus of the total force $|\nabla U|$ is very small and the Hessian of the molecular potential energy is positive.

Combining all terms, the elementary time propagator becomes

$$\{Q_{n+1}|\hat{U}(\Delta t)|Q_n\} \propto e^{-\frac{1}{2\sigma^2}(Q_{n+1}-Q_n)^2 - V_{\text{s}}\left(\frac{Q_n+Q_{n+1}}{2}\right)\frac{\Delta t}{\hbar_{\text{eff}}}}. \tag{S.22}$$

Some comments on this result are in order. First, we note that the elementary propagator is exponentially suppressed when the distance between Q_n and Q_{n+1} is larger than σ. On the other hand, any structure of $V_s(Q)$ below the short scale σ is smeared out due to the average involved in its definition (S.21). So, in the last term at the exponent we may use $V_{\text{s}}\left(\frac{Q_n+Q_{n+1}}{2}\right) \simeq V_{\text{s}}(Q_n)$, which yields

$$\{Q_{n+1}|\hat{U}(\Delta t)|Q_n\} \propto e^{-\frac{1}{2\sigma^2}(Q_{n+1}-Q_n)^2 - \frac{1}{2Dm}V_{\text{s}}(Q_n)\Delta t}, \tag{S.23}$$

where we have re-expressed the effective Dirac's constant in terms of the diffusion coefficient, $\hbar_{\text{eff}} = 2Dm$. Equation (S.23) qualitatively resembles the structure of the elementary propagator in the microscopic theory,

$$\langle x_{n+1}|\hat{U}(dt)|x_n\rangle \propto e^{-\frac{1}{4Ddt}(x_{n+1}-x_n)^2 - \frac{1}{2Dm}V_{\text{eff}}(x_n)dt}. \tag{S.24}$$

However, it is important to note that the time discretization step dt, required to obtain the propagator in the microscopic theory, is in general orders of magnitude smaller than that of the coarse-grained theory, Δt.

The coarse-grained propagator (S.23) can be cast in a form that is completely analog to the microscopic expression (S.24) by introducing the effective diffusion coefficient of the coarse-grained theory D_{cg}, defined in terms of the spatio-temporal resolution scales:

$$D_{\text{cg}} = \sigma^2/(2\Delta t). \tag{S.25}$$

We obtain

$$\{Q_{n+1}|\hat{U}(\Delta t)|Q_n\} \propto e^{-\frac{1}{4D_{\text{cg}}\Delta t}(Q_{n+1}-Q_n)^2 - \frac{1}{2D_{\text{cg}}m}V_{\text{cg}}(Q_n)\Delta t}, \tag{S.26}$$

where

$$V_{\text{cg}}(Q) = \frac{D_{\text{cg}}}{D}V_{\text{s}}(Q) \tag{S.27}$$

is the effective potential of the coarse-grained theory.

The re-scaling of the diffusion coefficient reflects the fact that the characteristic time scales of the coarse-grained theory are different from those of the original microscopic theory. To appreciate the physical origin of the rescaling, it is instructive to compute the probability for the system initially at some given position, to remain within an infinitesimal volume, after an infinitesimal time interval. To remove the dependence on normalization factors, it is convenient to compute this probability relative to the same quantity in the purely diffusive limit (i.e. for free Brownian motion). In the microscopic theory (using Eq. (S.24)) we obtain:

$$R(x,dt) \equiv \frac{\langle x|e^{-\left(\hat{T}_{\rm eff}+\hat{V}_{\rm eff}\right)\frac{dt}{\hbar_{\rm eff}}}|x\rangle}{\langle x|e^{-\hat{T}_{\rm eff}\frac{dt}{\hbar_{\rm eff}}}|x\rangle} = e^{-\frac{V_{\rm eff}(x)dt}{2Dm}}. \tag{S.28}$$

Thus, the ratio $V_{\rm eff}(x)/(2Dm)$ is identified with a rate of escape from an infinitesimal volume centered around Q.

The same calculation can be performed in the coarse-grained theory, using Eq. (S.26):

$$R(Q,\Delta t) \equiv \frac{\{Q|e^{-\left(\hat{T}_{\rm eff}+\hat{V}_{\rm eff}\right)\frac{\Delta t}{\hbar_{\rm eff}}}|Q\}}{\{Q|e^{-\hat{T}_{\rm eff}\frac{\Delta t}{\hbar_{\rm eff}}}|Q\}} = e^{-\frac{V_{\rm cg}(Q)\Delta t}{2mD_{\rm cg}}}. \tag{S.29}$$

In this low-resolution theory, $V_{\rm cg}(Q)/(2mD_{\rm cg})$ is interpreted as the rate of escape from a region of size σ centered in Q.

Even though the direct evaluation of $V_{\rm s}(Q)$ (and therefore of $V_{\rm cg}(x)$) is computationally very challenging, we can consider approximations to make it feasible in practice. For example, it could be directly estimated by computing the rate of escape from a region of size σ centered around each coarse-grained point $Q_i \in \mathscr{C}$, using the short MD simulations generated during the iMapD exploration.

In our first exploratory application, we choose to adopt a simpler phenomenological approach: we first smeared out the short-distance structure of $V_{\rm eff}$ through a self-averaging over the values of the effective potential evaluated on groups of structurally close configurations generated during the iMapD exploration. Let us denote the result of the average around point Q_i with $\langle V_{\rm eff}\rangle_{Q_i}$. Then, the effective potential of the coarse-grained theory $V_{\rm cg}(Q_i)$ was estimated by introducing a suitable renormalization constant, $V_{\rm cg}(Q_i) = \alpha\langle V_{\rm eff}\rangle_{Q_i}$.

In principle, the rescaling parameter α may be fixed in order to match global time scale, such as the average lifetime of the coarse-grained states, estimated by measuring how long it takes on average for the system to cover a distance σ in configuration space. In practice, since we were not interested in predicting time-dependent observables, the constant α was determined implicitly, by relying on the Hamilton–Jacobi formulation described in section S5.

S3: Fast exploration of alanine dipeptide's configuration space

S3.1: Exploring the intrinsic manifold

We start by sampling from two metastable states of alanine dipeptide in OpenMM. One starting from $C5$ region at the top left corner of Fig. S1, and from α_R in the vicinity of ($\phi = -75$,$\psi = -20$). After this initial sampling, we evaluate the diffusion map (DMAP) for each set of configurations separately to obtain a low-dimensional representation of the sampled configurations. We then identify the boundary in this representation, to initialize new simulations beyond the region that has already been sampled. We measure the pairwise distance between configurations by using the RMSD calculated on all non-hydrogen atoms after having removed global translations and rotations. The exploration proceeds by initiating unbiased sampling from each new configurations and then merging all the new data to the previous. By iterating over DMAP evaluation at every step, finding new configurations, and sampling we populate the transition region between $C5$ and α_R.

We eventually terminate the iterations when the configurations explored starting from the two initial metastable state overlap, i.e., when at least two configurations have RMSD closer than 0.3 Å (Fig. S2(a)).

S3.2: Simulation details

In all simulations, the molecule was placed in a square box with 2.85 nm base vector, solvated with the TIP3P water model using AMBER99SB forcefield[9]. We energy-minimized the initial configuration using L-BFGS algorithm implemented in OpenMM[10], with tolerance on the square mean root of all force components at 500 kJ (nm mole)$^{-1}$. Simulations were performed at a temperature $T = 300$ K, using a Langevin integrator with friction coefficient $\gamma = 91$ ps^{-1} and timesteps of $\Delta t = 2$ fs. The initial sampling bursts in the two metastable states were 200 ps long starting in $C5$ state and 20 ps for α_R. All following runs were 1 ps long.

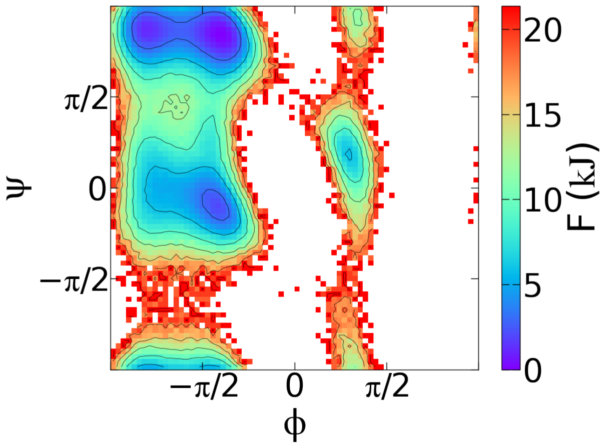

Figure S1. Free energy Landscape of alanine dipeptide, projected onto its two main dihedral angles. This figure was generated by simulating alanine dipeptide for 1 μs at $T = 300$ K, and in explicit TIP3P water. Contour lines are drawn every 3 kJ.

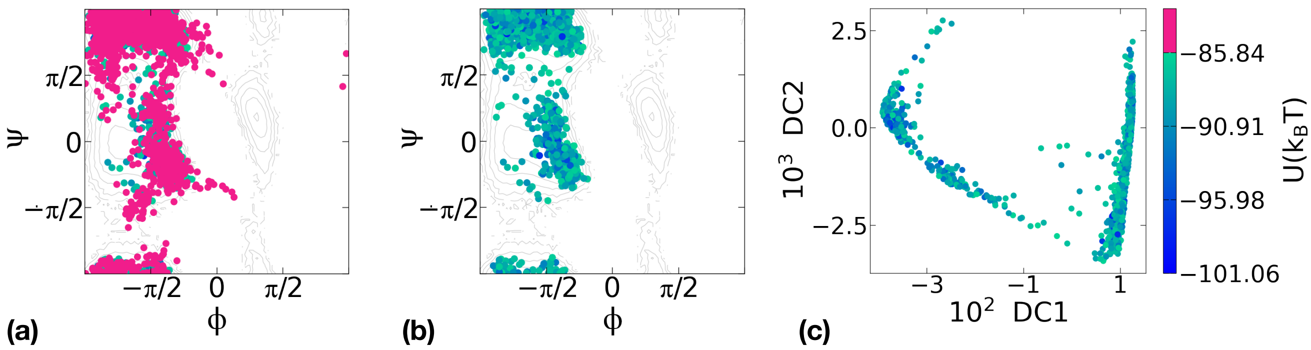

Figure S2. (a) Final result of the exploration with iMapD. (b) We further remove those points with higher potential energy than the median ($\approx$ 85.84 $k_\mathrm{B}T$), shown as dark pink color. (c) DMAP embedding of points whose potential is below median, shown as a function of the first two DCs.

S4: Graph Representation of the coarse-grained dynamics for alanine dipeptide

S4.1: Data Reduction and Identification of the Nodes in the Graph

Successive rounds of explorations carpeted the transition region between the basins associated with the α_R and $C5$ states. The number of sampled configurations needed to be reduced to obtain a sparser transition network compatible with the D-Wave quantum annealer. We first removed all configurations having a potential energy higher than the median value 85.84 $k_\mathrm{B}T$ (Fig. S2(b)). Second, we calculated the DMAP of all remaining samples and projected them on the first two diffusion coordinates (DCs), obtaining a set of points $\mathscr{C}_\mathrm{ini} = \{z_i\}$ (Fig. S2(c)). We then proceeded by identifying the two configurations in $\mathscr{C}_\mathrm{ini}$ with minimum RMSD distance from the initial configurations lying in two basins $C5$ (denoted by Q_A) and α (denoted by Q_B). Starting from z_A (corresponding to Q_A), we kept the nearest point z_{k1} satisfying $D_\mathrm{diff}(z_{k1}, z_A) > D_\mathrm{diff}^\mathrm{thresh}$, and removed from $\mathscr{C}_\mathrm{ini}$ all the configurations with lower D_diff. Here D_diff (diffusion distance) is the Euclidean distance between points in DMAP space, defined here as the plane spanned by the two diffusion coordinates DC1 and DC2. We used $D_\mathrm{diff}^\mathrm{thresh} = 7.5 \times 10^{-4}$ inspecting the histogram of nearest-neighbour pairwise diffusion distances among the points in $\mathscr{C}_\mathrm{ini}$, Fig S3(a). This value ensures that for any configuration in the original data set only one representative is kept, and henceforth their nearest neighbors based on D_diff—considered kinetically similar—are removed. We continued by applying the same procedure between remaining configurations in $\mathscr{C}_\mathrm{ini}$ and z_{k1}, and therefore identifying z_{k2}. By iterating over this step until there was no remaining element in $\mathscr{C}_\mathrm{ini}$, and finally appending back z_B to $\mathscr{C}_\mathrm{ini}$ (in case it was removed in previous iterations) we obtained a reduced version of our data set, $\mathscr{C}$, containing 83 points. $\mathscr{C}$ approximately span uniformly the DMAP embedding of the transition between the two metastable states (Fig. S3(b)-(c)).

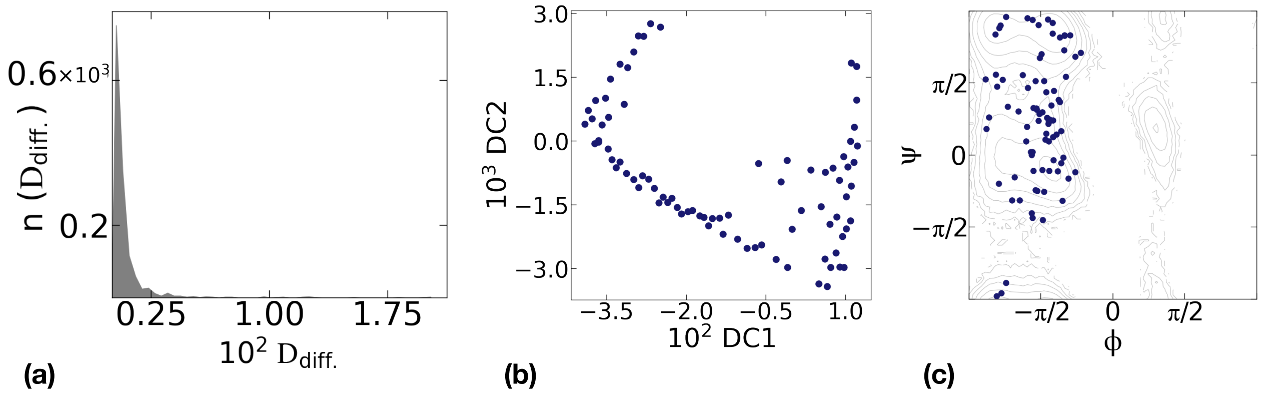

Figure S3. Sub-sampling explored configurations in a kinetically uniform way (a)D_{diff} histogram for nearest neighbors. $D_{\mathrm{diff}}^{\mathrm{thresh}} = 7.5 \times 10^{-4}$ contains more than 98% of nearest neighbor pairwise distances. Reduced data projected on (b) the first two DCs and (c) Ramachandran plot.

S4.2: Building the Graph

The next step required to build a graph having as nodes the sparse set of configurations $\mathscr{C}$ (Fig. S4). In this graph, only nodes who correspond to configurations that are kinetically and structurally close, should be connected. We used two criteria to insure this condition. Two nodes should be connected if their diffusion distance is smaller than 0.01 and their RMSD closer than 0.8 Å. Both thresholds were chosen heuristically from the histogram of pairwise diffusion distances and RMSD calculated on the set of configurations generated by iMapD (Fig. S5).

Num. of attempts	Correct topology	Wrong topology	Success rate
117	69	48	0.59

Table S1: Summary of the transition path generation on D-Wave to calculate the histogram reported in Fig. 4 of the main text

	Monte Carlo steps	Accepted paths	Wrong topology	Rejected paths
Markov chain 1	9	7	0	2
Markov chain 2	13	8	2	3
Markov chain 3	20	10	4	6

Table S2: Summary of the Markov chains sampling process on D-Wave.

S5: Details on the Calculation of the Graph Weights and the Determination of the Rescaling Parameter α

In the previous section, we have derived a suitable definition of the graph weights w_{ij} that explicitly depends on the time resolution Δt of our coarse-grained representation of the dynamics.

An alternative way of fixing a time resolution is to consider the Laplace transform of the elementary propagator,

$$G(Q_f, s|Q_i) = \int_0^\infty d\tau e^{-s\tau} K(Q_f, \tau|Q_i, 0). \tag{S.30}$$

In this expression, the time resolution is determined by the inverse of the frequency cut-off scale s. We adopted this regularization prescription in our illustrative application to alanine dipeptide.

To evaluate the elementary propagator in Laplace space, we resorted to the so-called Dominant Reaction Pathway (DRP) formalism[4,11], that originates from analyzing the path integral expression of the Feynman propagator $K(Q_f, t|Q_i, 0)$ in saddle-point approximation.

The saddle-point condition yields a Newton-type equation of motion for the most statistically relevant trajectory $\bar{Q}(\tau)$,

$$m\ddot{\bar{Q}} = \nabla V_{\mathrm{eff}}(\bar{Q}), \tag{S.31}$$

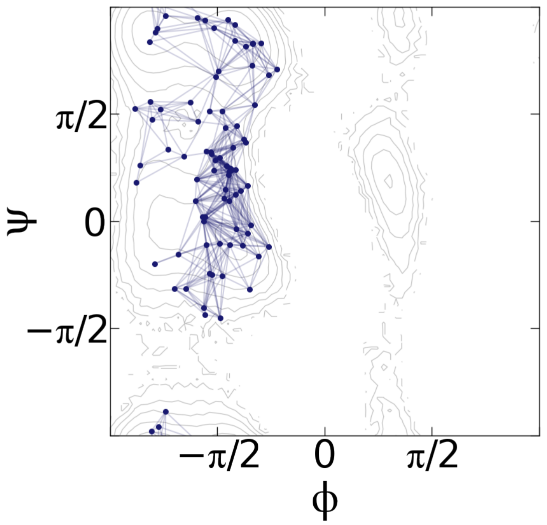

Figure S4. Network of configurations plotted on the Ramachandran plot. Nodes correspond to reduced data set $\mathscr{C}$, and edges connect configurations that are kinetically and structurally close. The number of nodes and edges are $\nu = 83$ and $|\mathscr{E}| = 495$, respectively.

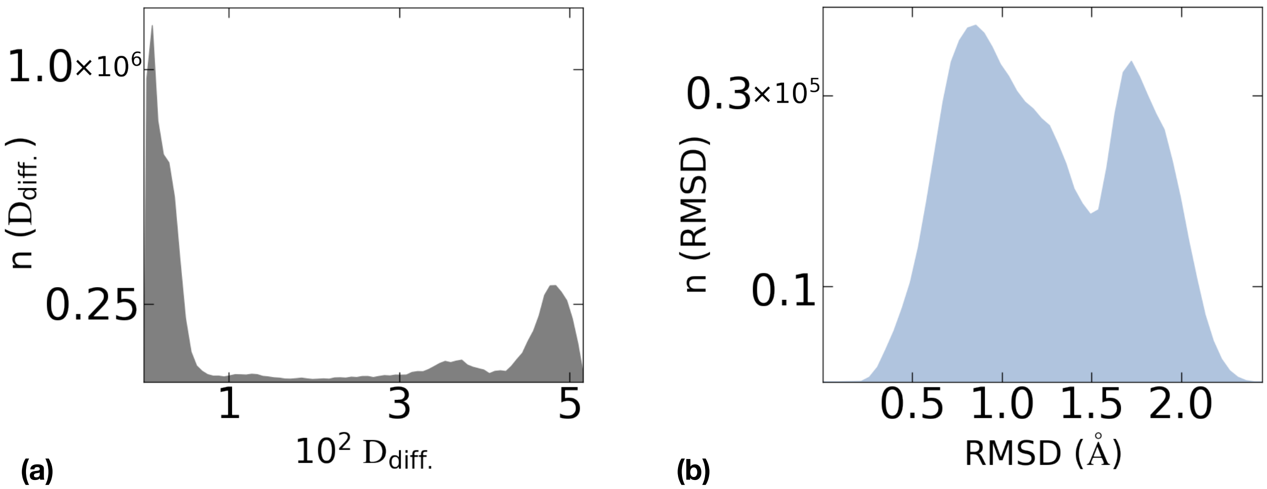

Figure S5. (a) Histogram of pairwise D_{diff} (b) and of pairwise RMSD calculated on all configurations sampled by iMapD.

where $\overline{Q}(\tau)$ obeys the boundary conditions $\bar{Q}(t) = Q_j$ and $\bar{Q}(0) = Q_i$.

Equation (S.31) implies the conservation of the effective energy

$$E_{\mathrm{eff}} = \frac{\dot{Q}^2}{2} m - V_{\mathrm{eff}}(Q) \tag{S.32}$$

along the most probable path, $\bar{Q}(\tau)$.

Using the conservation of E_{eff} and recalling the definition $\hbar_{\mathrm{eff}} = 2\frac{k_{\mathrm{BT}}}{\gamma}$, the Feynman path integral is expressed as

$$P(Q_f, t|Q_i) \simeq \mathscr{N}\, e^{-\frac{1}{2k_{\mathrm{B}}T}\left(U(Q_f)-U(Q_i)\right)}\, e^{\frac{\gamma}{2k_{\mathrm{B}}T}\left(E_{\mathrm{eff}}t - S_{HJ}[\bar{Q}]\right)}, \tag{S.33}$$

where $\mathscr{N}$ is an irrelevant normalization factor, $S_{\mathrm{HJ}}[\bar{Q}]$ is the so-called Hamilton–Jacobi (HJ) functional,

$$S_{\mathrm{HJ}}[Q] = \int_{Q_i}^{Q_f} dl \sqrt{2m\left(E_{\mathrm{eff}} + V_{\mathrm{eff}}[\bar{Q}(l)]\right)}, \tag{S.34}$$

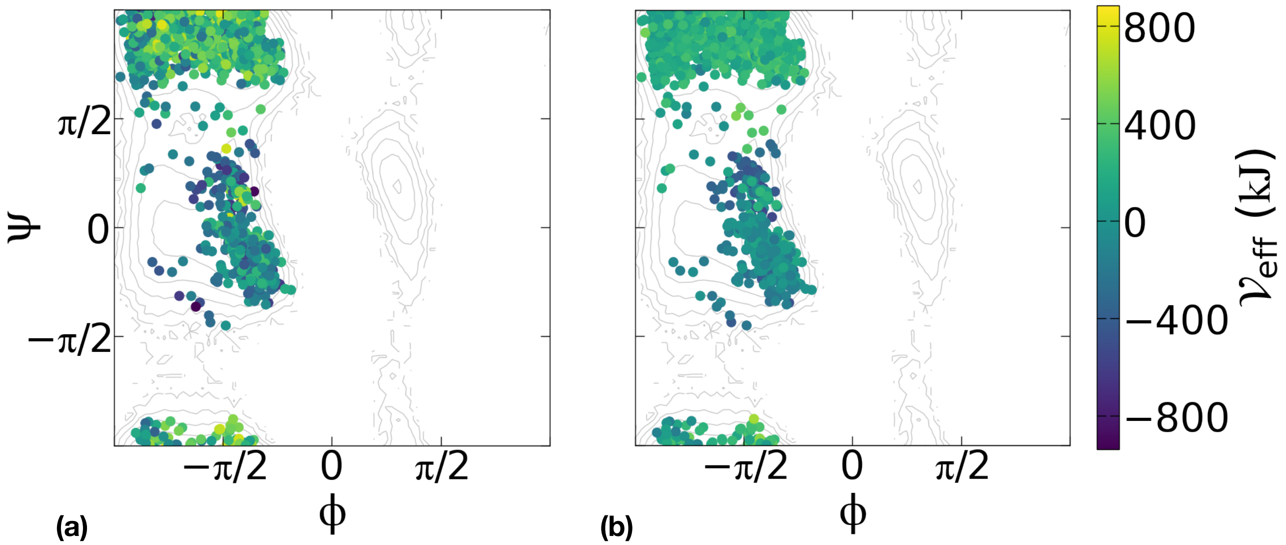

Figure S6. The original V_{eff} (a) and averaged version (b). In order to average V_{eff}, we used the embedding of configurations on first two DCs, and averaged over configurations who were close to each other.

and dl is the Euclidean distance travelled along the saddle-point trajectory $\bar{Q}$.

Using Eq. (S.33), and recalling that $st \gg 1$, the Laplace time integral, Eq. (S.30), finally becomes:

$$G(Q_f, s|Q_i) = \mathcal{N} e^{-\frac{1}{2k_B T}\left(U(Q_f)-U(Q_i)\right)} e^{-\frac{\gamma}{2k_B T}\int_{Q_i}^{Q_f} dl \sqrt{2m(s+V_{\text{eff}}[\bar{Q}(l)])}}. \tag{S.35}$$

We note that the exponential pre-factor can be ignored when comparing the probability density of transition paths connecting the same initial and final configuration. Therefore, in the graph representation, we can retain only the second exponent.

Equation (S.35) can be used to obtain the weights of the links in our coarse-grained graph representation of the Langevin dynamics. We obtain

$$w_{ij} = \frac{|Q_i - Q_j|}{2\sqrt{D_{\text{cg}}}}\left(L_i + L_j\right), \quad L_i = \sqrt{V_{\text{cg}}(Q_i) + s_0}\,, \tag{S.36}$$

where s_0 is a cut-off time scale, estimated from the value of the effective potential in the reactant state Q_i, i.e. $s_0 \sim -V_{\text{cg}}(Q_i)$ (for a detailed discussion on this point, see[11]).

We note that an advantage of the frequency regularization (relative to the conventional time regularization) is that the resulting expression for w_{ij} depends on the Hamilton–Jacobi functional, which is defined in terms of structural distances Δl between configurations. The convenience of this discretization arises from the fact that there is no gap in the internal distance scales of molecular systems.

We recall that, in the present first application, we estimated the coarse-grained effective potential using $V_{\text{cg}}(Q) = \alpha \langle V_{\text{eff}} \rangle_Q$, where $\langle \cdot \rangle_Q$ denotes a local average performed over the configurations near Q generated during iMapD exploration. Then,

$$L_i = \sqrt{\alpha \langle V_{\text{eff}} \rangle_{Q_i} + s_0}, \tag{S.37}$$

where $s_0 \sim -\alpha \langle V_{\text{eff}} \rangle_{Q_i}$.

As discussed in the previous section, α could be in principle determined by matching some global time scale computed from microscopic simulations. In this work, we adopted a simpler approach and eliminated the dependence on this parameter by re-normalizing all weights w_{ij}, by dividing them by the largest link in the graph $w_{\max}$. This procedure ensured that the relative probabilities of the paths in the graph are comparable and finite, as they should in the coarse-grained theory.

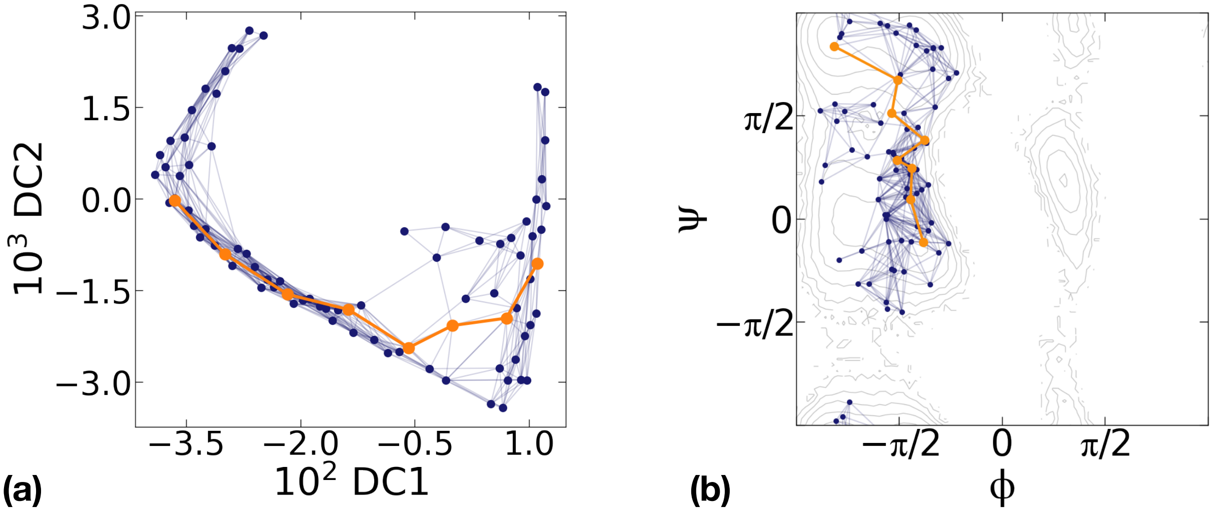

Figure S7. The most probable path, calculated via Dijkstra algorithm, in (a) DMAP embedding and (b) Ramachandran plot.

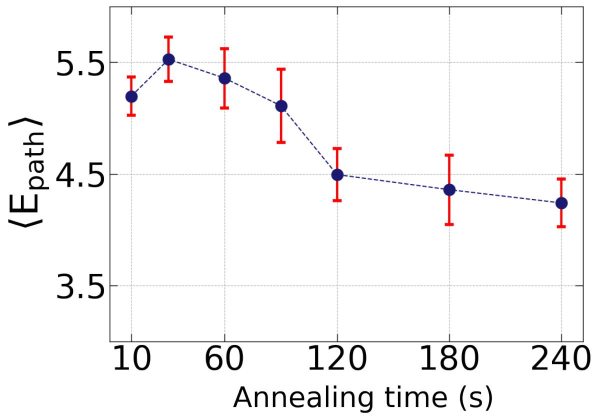

Figure S8. Average value and standard error of the mean of the energy E obtained by multiple quantum annealing processes at fixed values of t_{sweep}. These results are used to estimate $P(E|t_{\text{sweep}})$ to lowest order in the cumulant expansion approximation.

S6: Auto-Correlation function

The function $G(N)$ used to estimate the auto-correlation along the Markov chain of our hybrid Monte Carlo algorithm is defined as follows,

$$G(N) = \frac{1}{N_{\text{MC}}}\sum_{k=1}^{N_{\text{MC}}}\left[\left\langle \frac{1}{|\mathscr{E}|}\sum_{i<j}{}' \Gamma^{(2)}_{ij}(k+N)\Gamma^{(2)}_{ij}(k)\right\rangle - \left\langle \frac{1}{|\mathscr{E}|}\sum_{i<j}{}' \Gamma^{(2)}_{ij}(k+N)\right\rangle\left\langle \frac{1}{|\mathscr{E}|}\sum_{i<j}{}' \Gamma^{(2)}_{ij}(k)\right\rangle\right]. \tag{S.38}$$

In this equation, the $\Gamma^{(2)}(k)$ represent the collection of all binary link variables generated at the k-th Monte Carlo step and we are implicitly assuming periodic boundary conditions, i.e., $\Gamma^{(2)}_{ij}(N_{\text{MC}}+1) = \Gamma^{(2)}(1)_{ij}$. Here, N_{MC} is the number of Monte Carlo steps, $\sum'$ denotes a summation restricted to the ε pairs of indexes i and j that label topologically adjacent sites in the graph and $|\mathscr{E}|$ denotes the number of edges present in the graph. Finally, N is the distance in the Monte Carlo chain. The average $\langle\cdot\rangle$ is intended over many Monte Carlo trajectories. In practice, however, the computing time that was available to us on the D-Wave quantum computer was sufficient to generate only 3 Monte Carlo trajectories. Since such a limited statistics does not allow us to estimate the averages in Eq. (S.38), we chose not to perform the average and directly analyze the behavior of $G(N)$ for each independent trajectory.

Due to the absence of autocorrelation, the cost of generating an ensemble of N independent transition paths scales like $\sim \frac{Ns}{a}$, where s is the cost of a single Monte Carlo step and a is the average acceptance ratio. In our case, s amounts to about 120

seconds of total computing (of which about 6 seconds of quantum computing) and $a \simeq 0.6$. Therefore, to produce an ensemble of about 100 independent transition paths for this system, our Monte Carlo scheme would require slightly more than 5 hours of total computing time, including about 15 minutes of QPU time.

References

1. Chiavazzo, E. et al. Intrinsic map dynamics exploration for uncharted effective free-energy landscapes. Proc. Natl. Acad. Sci. **114**, E5494–E5503, DOI: 10.1073/pnas.1621481114 (2017).
2. Coifman, R. R. & Lafon, S. Diffusion maps. Appl. Comput. Harmon. Analysis **21**, 5–30, DOI: 10.1016/j.acha.2006.04.006 (2006).
3. Elber, R. & Shalloway, D. Temperature dependent reaction coordinates. The J. Chem. Phys. **112**, 5539–5545, DOI: 10.1063/1.481131 (2000).
4. Faccioli, P., Sega, M., Pederiva, F. & Orland, H. Dominant pathways in protein folding. Phys. Rev. Lett. **97**, DOI: 10.1103/physrevlett.97.108101 (2006).
5. Eastman, P., Grønbech-Jensen, N. & Doniach, S. Simulation of protein folding by reaction path annealing. The J. Chem. Phys. **114**, 3823–3841, DOI: 10.1063/1.1342162 (2001).
6. Onsager, L. & Machlup, S. Fluctuations and irreversible processes. Phys. Rev. **91**, 1505–1512, DOI: 10.1103/physrev.91.1505 (1953).
7. Caroli, B., Caroli, C. & Roulet, B. Diffusion in a bistable potential: The functional integral approach. J. Stat. Phys. **26**, 83–111, DOI: 10.1007/bf01106788 (1981).
8. Autieri, E., Faccioli, P., Sega, M., Pederiva, F. & Orland, H. Dominant reaction pathways in high-dimensional systems. The J. Chem. Phys. **130**, 064106, DOI: 10.1063/1.3074271 (2009).
9. Lindorff-Larsen, K. et al. Improved side-chain torsion potentials for the amber ff99sb protein force field. Proteins: Struct. Funct. Bioinforma. **78**, 1950–1958, DOI: 10.1002/prot.22711 (2010).
10. Eastman, P. et al. OpenMM 7: Rapid development of high performance algorithms for molecular dynamics. PLOS Comput. Biol. **13**, e1005659, DOI: 10.1371/journal.pcbi.1005659 (2017).
11. Sega, M., Faccioli, P., Pederiva, F., Garberoglio, G. & Orland, H. Quantitative protein dynamics from dominant folding pathways. Phys. Rev. Lett. **99**, DOI: 10.1103/physrevlett.99.118102 (2007).
